# The Influence of Plasma and Peritoneal Antiretroviral Drugs Levels on the Shedding of HIV‐1 Into Peritoneal Dialysis Effluent

**DOI:** 10.1002/jmv.71192

**Published:** 2026-10-24

**Authors:** Teboho Mooko, Feziwe Bisiwe, Perpetual Chikobvu, Molefi Morobadi, Martin Nyaga, Dominique Goedhals, Thabiso Mofokeng, Gabre Kemp, Kwazi Ndlovu

**Affiliations:** ^1^ Department of Internal Medicine University of the Free State Bloemfontein South Africa; ^2^ Next Generation Sequencing Unit and Division of Virology University of the Free State Bloemfontein South Africa; ^3^ Division of Nephrology University of the Free State Bloemfontein South Africa; ^4^ Department of Health of the Free State Bloemfontein South Africa; ^5^ Department of Community Health University of the Free State Bloemfontein South Africa; ^6^ Division of Virology University of the Free State Bloemfontein South Africa; ^7^ Ampath Laboratories Pretoria South Africa; ^8^ PathCare Vermaak Pretoria South Africa; ^9^ Department of Microbiology and Biochemistry University of the Free State Bloemfontein South Africa; ^10^ Milnerton Mediclinic Cape Town South Africa

**Keywords:** antiretroviral‐therapy, HIV‐shedding, kidney‐failure, peritoneal‐dialysis

## Abstract

Penetrance of antiretroviral therapy in the peritoneal cavity and its influence on the shedding of HIV‐1 into continuous ambulatory peritoneal dialysis (CAPD) was investigated. This prospective cross‐sectional study of HIV‐positive end‐stage kidney failure (ESKF) patients managed with ART and CAPD, collected enrolled patients' demographic information, clinical and laboratory data. ART levels were quantified using liquid chromatography coupled to mass spectrometry. There were 60 patients recruited with a median age of 43.0 (IQR: 38.0–47) years and were predominantly on abacavir (88.3%) lamivudine (98.3%) and efavirenz (70.0%) for a median duration of 8 (IQR: 5–11) years. Participants with detectable HIV‐1 had lower abacavir (2.10 vs. 8.15 ng/µL; *p* = 0.054), lamivudine (0.018 vs. 1.065 ng/µL; *p* = 0.003) and efavirenz (3.605 vs. 57.1 ng/µL; *p* = 0.012) plasma levels compared to those with undetectable HIV‐1 in CAPD effluents. Measured lamivudine (1.065 vs. 0.888 ng/µL; *p* = 0.001), abacavir (8.15 vs. 4.95 ng/µL; *p* = 0.024), efavirenz (57.1 vs. 3.65 ng/µL; *p* < 0.001) and dolutegravir (2.68 vs. 0.65 ng/µL; *p* < 0.001) drug levels were significantly higher in plasma than CAPD effluents. The peritoneal to plasma (D/P) ratios of these ART agents was highly variable. On Spearman's rank correlation test, plasma levels of lamivudine (*p* = 0.003), abacavir (*p* < 0.035) and efavirenz (*p* = 0.008) were significantly correlated with HIV‐1 shedding into CAPD effluents. The findings suggest that low plasma ART drug levels were strongly associated with HIV‐1 shedding into CAPD effluents. Moreover, there was an inverse correlation between plasma drug concentration of lamivudine, efavirenz, and abacavir and the HIV‐1 viral load detectable in CAPD effluents.

## 1 Introduction

HIV is associated with an increased risk of chronic kidney diseases (CKD) and progression to end‐stage renal failure (ESKF) at a younger age [1]. A tenofovir‐sparing regimen that includes dolutegravir, abacavir, and lamivudine is a common ‘renal‐friendly’ antiretroviral therapy (ART) regimen recommended by most clinical practice guidelines in people living with HIV (PLWHIV) and have CKD [2, 3]. Renal‐friendly ART agents are rapidly absorbed and highly bioavailable following oral administration and are widely distributed throughout the body [4, 5, 6]. A total of 70% of lamivudine is excreted unchanged through the kidneys. Thus, it requires renal adjustment in CKD patients [3]. The adjusted dosages of lamivudine are thought to provide sufficient viral suppression comparable to non‐kidney disease PLWHIV [7, 8, 9]. However, ART inter‐ and intra‐patient pharmacokinetic variability, poor adherence, long‐term exposure, and several other factors have been reported to contribute to sub‐therapeutic ART levels and treatment failure [10]. Furthermore, effective ART penetrance and levels in HIV‐1 reservoir sites such as, central nervous system, genital tract and peritoneal cavity are critical determinants of ART effects and outcomes [11, 12]. Routine monitoring of ART levels is currently controversial but may find value in cases where poor virologic and immunological failure is observed [10].

HIV‐1 shedding into CAPD effluents of PLWHIV and have ESKF has been reported in individuals on a full complement of ART [13, 14, 15]. This was associated with the presence of HIV drug resistance mutations (HIVDRMs) in a recent study by Mooko et al. [16]. The contamination of CAPD waste and equipment raises concerns for the immediate contacts of these patients and could be a strong predictor of ART failure [13, 14]. There is a lack of research data elucidating the penetrance of ART into the peritoneal cavity of HIV positive ESKF patients with detectable HIV‐1 particles in CAPD effluents. Thus, the aim of the study was to determine ART drug penetration in the peritoneal compartment and its effect on the shedding of HIV‐1 into CAPD effluents.

## 2 Materials and Methods

### 2.1 Study Population

This prospective cross‐sectional study recruited consecutive prevalent and incident PLWHIV and having ESKF and managed with CAPD at Universitas Academic Hospital in Bloemfontein, South Africa. Patient recruitment and sample collection was carried out over a 2‐year period, from the 31st April 2021 until the 31st April 2023. All participants provided written consent prior to enrollment, and study procedures were done in accordance with the Helsinki Declaration of clinical research on human subjects [17]. Patients with peritonitis were excluded from the study. The study protocol was approved by the University of the Free State‐Health Sciences Research Ethics Committee (UFS‐HSREC) (UFS‐HSD2020/0318/2710 & UFS‐HSD2021/0267/2505), and permission for the study was granted by the Free State Department of Health. HIV status and ART were confirmed by the patients' local clinic prior to enrollment as per South African HIV diagnostics and ARV guidelines [2]. Y‐sets, twin‐bag systems, and conventional peritoneal dialysis (PD) solutions (Adcock Ingram and Fresenius, South Africa) were used in all CAPD patients as described previously [16].

### 2.2 Patient Enrollment

Patient's demographic information, anthropometric measurements, vital signs and PD fluid and blood samples were collected at enrollment. A 4‐h‐timed peritoneal equilibration test (PET) was performed on a scheduled date suitable for both the research nurse and the patient. Information on Laboratory tests for full blood counts, T‐cell CD4 count, serum levels of urea, creatinine, electrolytes, eGFR, ferritin, and albumin were periodically retrieved from the Universitas Academic Hospital electronic results database.

### 2.3 Sample Collection, Preparation, and Storage

At enrollment, a research nurse collected 4 mL blood samples, using BD K3EDTA and serum separation vacutainers (Lasec, South Africa) and disposable needles, and CAPD effluent samples, using a sterile 10 mL syringe, directly from a functioning Tenckhoff catheter into a sterile specimen bottle. The first 10 mL of CAPD effluent was aspirated and discarded. All samples were transported on ice to the research laboratory, where whole blood was centrifuged at 2500 rpm for 15 min, and plasma as well as serum were separated in hooded benchtops. Both separated plasma, serum, cellular component, and aliquoted CAPD effluent samples were freeze‐stored in 2 mL Eppendorf tubes at −70°C throughout the study period, and later used for ART levels quantification and HIV viral load analysis.

To determine the peritoneal membrane type of the patient, a 2.5% dialysate solution was used during PET. CAPD effluent samples were collected from functioning CAPD catheters at times 0, 2, and 4 h, along with corresponding plasma and serum samples. A Supporting Information S1: File on PET procedures, CAPD sample collection and peritoneal membrane type calculation is attached.

### 2.4 Sample Preparation Through Solid Phase Extraction

This procedure was performed as reported in our previous study [18]. Analytes (including spiked 20 µL of 0.1 ppm abacavir‐d4 (Internal Standard)) from 500 µL plasma and PD effluents were extracted, desalted, and concentrated onto methanol‐conditioned C_18_ 1 mL solid phase extraction cartridges (Oasis Prime HLB 1cc/30 mg, Waters) at a flow rate of 1 mL/min. Bound analytes were slowly eluted off the dried cartridges using 1 mL of methanol. The eluent was vacuum dried using Savant Speedvac (ThermoFisher Scientific, South Africa) until almost dry, and reconstituted in a 500 µL H_2_O with 0.1% formic acid.

### 2.5 Liquid Chromatography Analysis and Conditions

All standard drug samples were analyzed using an ABSCIEX 4000 QTRAP hybrid triple quadrupole ion trap mass spectrometer (AB SCIEX, Framingham, MA, USA) with a Shimadzu HPLC (Shimadzu, Roodepoort, South Africa) stack as a front end. 20 µL of each extracted sample was separated on a C_18_ (50 × 2.0 mm, Aqua, Phenomenex) column at a flow rate of 300 µL/min using a 4 min gradient from 45% solvent A (water in 0.1% formic acid) to 95% solvent B (methanol in 0.1% formic acid) with a total run time of 15 min to allow for column re‐equilibration [18].

The nominal concentrations ranged from 0.005 to 1.25 ppm for the abacavir calibration curve using 500 µL sample volume. The intra‐assay precision and accuracy of the method (coefficient of variation) were 9.1% and 10.6%, respectively, while at the lowest limit of quantification, it was 14.6% and 16.3%, respectively. Chromatogram of each ARV standard drug is presented in Supporting Information S1: Figure S1, and the mass spectrometry conditions summarized in Supporting Information S1: Table S1.

### 2.6 Statistical Analysis

Continuous variables were summarized as medians and interquartile ranges (IQR) and compared using Wilcoxon rank‐sum (Mann‐Whitney) test. Categorical and ordinal variables were summarized using proportions and percentages (95% confidence interval). These were compared using Pearson's Chi‐square test or Fisher's exact test, as appropriate. Spearman rank correlation test was used to measure the association of clinical, biochemical and laboratory variables with the detection of HIV‐1 in CAPD effluents. All analyses were performed using Stata version 15 (StataCorp LP, College Station, TX, USA). The level of significance was set at *p* < 0.05.

## 3 Results

### 3.1 Patient Characteristics

The study enrolled 60 patients with a median age of 43.0 (IQR: 38.0–47) years, and 71.7% (43/60) were female. The majority of participants were managed with an oral daily dosage of 50 mg lamivudine (98.3%; 59/60), 600 mg abacavir (88.3%; 53/60), and 600 mg efavirenz (70%; 42/60), 18.3% (11/60) were on dolutegravir, among others. The median duration of ARV treatment at the time of enrollment was 8 (IQR: 5–11) years (~417 weeks), and CAPD treatment was 9.5 (IQR: 3.7–26.5) months (~41 weeks). The median T‐cell CD4 count was 328 (IQR: 238–490) cells/mm^3^. The majority of the Tenckhoff catheters were inserted laparoscopically by surgeons (*n* = 25), while a few (*n* = 35) percutaneous Tenckhoff catheters were inserted by a nephrologist at Universitas Academic Hospital. There were 8 participants (13.3%; 95% CI: 6.7%–24.8%) who had detectable HIV‐1 particles in CAPD effluents with a median viral load of 35.2 (IQR: 30.6–213.1) copies/mL measured in CAPD effluents. Table 1 summarizes demographic and clinical characteristics of the study population according to the detection of HIV‐1 in CAPD effluents.

**Table 1:** Demographic and clinical characteristics of the study population.

	HIV‐1 in PD effluents		*p*‐value	Total (*N* = 60)
	Undetectable (*N* = 52)	Detectable (*N* = 8)		
Demographics				
Age (years), median (IQR)	42.4 (38.5–48.7)	43.7 (33.8–44.6)	0.656	43.0 (38.0–47.0)
Sex				
Female, n/N (%)	37/52 (71.2%)	6/8 (75.0%)	1.00	43/60 (71.7%; 58.8%–81.8%)
Ethnicity				
African, n/N (%)	52/52 (100.00%)	8/8 (100.00%)		60/60 (100%)
Marital status				
Married, n/N (%)	13/52 (25.0%)	2/8 (25.0%)	1.00	8/60 (25.0%; 15.5%–37.7%)
Single, n/N (%)	39/52 (75.0%)	6/8 (75.0%)		30/60 (75.0%; 62.3%–84.5%)
Employment status				
Employed, n/N (%)	4/52 (7.69%)	0/8 (0.00%)	1.00	4/60 (6.7%; 2.5%–16.8%)
Unemployed, n/N (%)	48/52 (92.3%)	8/8 (100%)		56/60 (93.3%; 83.2%–97.5%)
Residential area				
Rural, n/N (%)	19/52 (36.5%)	1/8 (12.5%)	0.249	20/60 (33.3%; 22.4%–46.4%)
Township, n/N (%)	33/52 (63.5%)	6/8 (87.5%)		40/60 (66.7%; 53.6%–77.6%)
Highest education				
Primary school, n/N (%)	8/52 (15.4%)	1/8 (12.5%)	1.00	9/60 (15.0%; 7.9%–26.7%)
High school, n/N (%)	29/52 (55.8%)	5/8 (62.5%)		34/60 (56.7%; 43.7%–68.8%)
Grade 12 certificate, n/N (%)	13/52 (25.0%)	2/8 (25.0%)		15/60 (25.0%; 15.5%–37.7%)
Degree, n/N (%)	2/52 (3.8%)	0/8 (0.00%)		2/60 (3.3%; 0.8%–12.7%)
Cigarette smoker, n/N (%)	4/52 (7.7%)	1/8 (12.5%)	0.339	5/60 (8.3%; 3.4%–18.8%)
Clinical characteristics				
Hypertension, n/N (%)	50/52 (96.2%)	8/8 (100.00%)	1.00	58/60 (96.7%; 87.3%–99.2%)
HIVAN, n/N (%)	19/52 (36.5%)	5/8 (62.5%)	0.247	24/60 (40.0%; 28.2%–53.0%)
CAPD duration (months), median (IQR)	9.5 (3.23–28.89)	12.4 (5.0–22.1)	0.811^a^	9.5 (3.7–26.5)
ARV duration (years), median (IQR)	8 (5–11)	7.5 (5–10)	0.702^b^	8 (5–11)
Systolic BP (mmHg), median (IQR)	152 (136–173.5)	155 (142.5–169)	0.802	152.5 (136–173.5)
Diastolic BP (mmHg), median (IQR)	98 (83.5–113.5)	102 (87.5–110)	0.777	98 (83.5–111.5)
BMI (Kg/m^2^), median (IQR)	24.7 (22.2–27.9)	23.6 (22.2–25.4)	0.485	24.3 (22.2–27.8)
Laboratory parameters				
HIV viral load^c^				
PD effluents, median (IQR), copies/mL		35.2 (30.6–213.1)	< 0.001	35.2 (30.6–213.1)
Plasma, median (IQR), copies/mL	79.15 (32.5–123)	34 550 (10040–86100)	< 0.001	116 (60–1980)
T‐cell CD4 count (cells/mm^3^), median (IQR)	437 (321–631)	293 (198–302)	0.006	328 (238–490)
CD4 count < 200 cells/mm^3^, n/N (%)	1/52 (1.9%)	2/7 (28.6%)	0.003	3/59 (5.1%; 1.6%–14.9%)
CD4 count 200–349 cells/mm^3^, n/N (%)	15/52 (28.8%)	4/7 (57.1%)		19/59 (32.2%; 21.4%–45.3%)
CD4 count ≥ 350 cells/mm^3^, n/N (%)	36/52 (69.2%)	1/7 (14.3%)		37/59 (62.7%; 49.5%–74.2%)
Haemoglobin (g/dL), median (IQR)	10.35 (9.3–12.3)	10.45 (9.3–11.35)	0.862	10.35 (9.3–12.1)
White blood cell count (× 10^9^ cells/L), median (IQR)	5.81 (5.01–7.44)	4.96 (3.81–5.39)	0.017	5.53 (4.83–7.24)
Sodium (mmol/L), median (IQR)	139 (136–142)	137 (135–139)	0.165	139 (136–142)
Potassium (mmol/L), median (IQR)	4 (3.4–4.4)	4.15 (3.8–5.85)	0.166	4 (3.4–4.4)
Urea (mmol/L), median (IQR)	20.35 (14.35–23.4)	19.2 (12.35–29.9)	0.922	19.9 (14–23.5)
Creatinine (µmol/L), median (IQR)	1021 (714–1272.5)	479 (326–719.5)	0.002	952 (679–1263)
Calcium(mmol/L), median (IQR)	2.17 (2.01–2.33)	2.03 (1.77–2.1)	0.053	2.16 (1.98–2.26)
Phosphate(mmol/L), median (IQR)	1.48 (1.06–1.76)	1.27 (1.09–1.32)	0.358	1.36 (1.07–1.7)
Albumin	28 (22–30)	20 (17–28)	0.102	28 (20–30)
Albumin ≥ 35, n/N (%)	1/52 (1.9%)	0/8 (0%)	1.000	1/60 (1.7%; 0.2%–11.3%)
Albumin 30–34.9, n/N (%)	16/52 (30.8%)	2/8 (25%)	1.000	18/60 (30.0%; 19.6%–43.0%)
Albumin 25–29.9, n/N (%)	14/52 (26.9%)	0/8 (0.0%)	0.179	14/60 (23.3%; 14.2%–35.9%)
Albumin < 25, n/N (%)	21/52 (29.0%)	6/8 (71.4%)	0.124	27/60 (45.0%; 32.7%–58.0%)
Ferritin	337 (182.5–593)	397 (293–407)	0.564	388 (211–605)
Peritoneal equilibration test				
D/P creatinine, median (IQR)	0.682 (0.602–0.727)	0.708 (0.643–0.793)	0.341^b^	0.690 (0.603–0.733)
D/D_0_ glucose, median (IQR)	0.496 (0.410–0.542)	0.448 (0.337–0.542)	0.595^b^	0.494 (0.395–0.542)
Ultrafiltration volume (mL), median (IQR)	300 (300–400)	300 (200–300)	0.327	300 (300–400)

Abbreviations: ARV, antiretroviral; BMI, body mass index; CAPD, continuous ambulatory peritoneal dialysis; D/D_0_, plasma to dialysate concentrations; D/P, peritoneal to plasma ratio; HIVAN, HIV‐associated nephropathy.

^a^ Pearson's *χ* ^2^ test.

^b^ Two‐sample Wilcoxon rank‐sum (Mann‐Whitney) test.

^c^ Median HIV‐1 viral load estimates were calculated using only samples with quantifiable viral loads above the assay's lower limit of detection.

### 3.2 ARV Drug Level Quantification and Peritoneal to Plasma Drug Levels Ratio

Participants with detectable HIV‐1 had lower abacavir (2.10 vs. 8.15 ng/µL; *p* = 0.054), lamivudine (0.018 vs. 1.065 ng/µL; *p* = 0.003) and efavirenz (3.605 vs. 57.1 ng/µL; *p* = 0.012) plasma levels compared to those with undetectable HIV‐1, Table 2. Measured lamivudine (1.065 vs. 0.888 ng/µL; *p* < 0.001), efavirenz (57.1 vs. 3.65 ng/µL; *p* < 0.001) and dolutegravir (2.68 vs. 0.65 ng/µL; *p* < 0.001) drug levels were significantly higher in plasma than CAPD effluents during the regular clinic and PET visit, Figure 1.

**Table 2:** ARV drug levels.

	HIV‐1 in PD effluents		*p*‐value	Total (*N* = 60)	*p*‐value
	Undetectable (*N* = 52)	Detectable (*N* = 8)			
Clinic visit					
Lamivudine, n/N (%)	51/52 (98.1%)	8/8 (100.0%)^a^	1.000^b^	59/60 (98.3%; 88.7%–99.8%)	
Plasma Lamivudine levels	1.065 (0.040–3.950)	0.018 (0.011–0.037)	0.003^c^	0.455 (0.021–3.33	< 0.001^d^
PD effluent Lamivudine levels	0.888 (0.043–2.765)	0.018 (0.007–0.032)	0.001^c^	0.342 (0.025–2.565)	
Lamivudine Peritoneal to Plasma ratio	0.771 (0.498–0.954)	0.603 (0.425–1.250)	0.479^c^	0.761 (0.478–0.954)	
Abacavir, n/N (%)	45/52 (86.5%)	8/8 (100.0%)	0.578	53/60 (88.3%; 77.2%–94.4%)	
Plasma Abacavir levels	8.15 (3.62–16.9)	2.10 (0.0008–4.82)	0.054	5.68 (1.88–16.5)	0.024 d
PD effluent Abacavir levels	4.95 (2.02–10.7)	2.83 (0.0083–12.35)	0.427	4.95 (1.76–11.4)	
Abacavir Peritoneal to Plasma ratio	0.644 (0.444–0.888)	1.717 (1.142–3.143)	0.027	0.674 (0.447–1.477)	
Efavirenz, n/N (%)	36/52 (69.2%)	6/8 (75%)	1.000	42/60 (70.0%; 57.0%–80.4%)	
Plasma Efavirenz levels	57.1 (21.15–80.95)	3.605 (1.47–27.7)	0.012	46.2 (11.7–75.9)	< 0.001^d^
PD effluent Efavirenz levels	3.65 (0.66–8.34)	0.665 (0.028–1.44)	0.020	2.345 (0.282–7.53)	
Efavirenz Peritoneal to Plasma ratio	0.090 (0.024–0.247)	0.048 (0.019–0.444)	0.943	0.084 (0.024–0.252)	
Dolutegravir, n/N (%)	10/52 (19.2%)	1/8 (12.5%)	1.000	11/60 (18.3%; 10.3%–30.4%)	
Plasma Dolutegravir levels	2.68 (0.139–8.01)	0.483 (0.483–0.483)	0.527	1.91 (0.139–8.01)	0.001^d^
PD effluent Dolutegravir levels	0.65 (0.081–5.26)	0.099 (0.099–0.099)	0.527	0.42 (0.081–5.26)	
Dolutegravir Peritoneal to Plasma ratio	0.653 (0.215–0.804)	0.204 (0.204–0.204)	0.343	0.629 (0.204–0.804)	
Lopinavir/Ritonavir, n/N (%)	7/52 (13.5%)	1/8 (12.5%)	1.000	8/60 (13.3%; 6.7%–24.7%)	
Plasma Lopinavir levels	14.9 (0.068–280)	0.053 (0.053–0.053)	0.127	7.631 (0.066–152.10)	0.727^d^
PD effluent Lopinavir levels	0.598 (0.431–13.7)	0.094 (0.094–0.094)	0.275	0.598 (0.262–11.24	
Lopinavir Peritoneal to Plasma ratio	0.566 (0.031–1.194)	1.778 (1.778–1.778)	0.275	0.712 (0.036–1.486)	
Plasma Ritonavir levels	2.450 (0.233–7.28)	0.323 (0.323–0.323)	0.513	1.820 (0.278–4.94)	0.008^d^
PD effluent Ritonavir levels	0.277 (0.066–0.49)	0.072 (0.072–0.072)	0.513	0.193 (0.069–0.48)	
Ritonavir Peritoneal to Plasma ratio	0.091 (0.025–0.282)	0.222 (0.222–0.222)	0.513	0.135 (0.031–0.252)	
Zidovudine, n/N (%)	6/52 (11.5%)	0/8 (0.0%)	0.585	6/60 (10.0%; 4.5%–20.8%)	
Plasma Zidovudine levels	0.842 (0.001–3.92)			0.842 (0.001–3.92)	
PD effluent Zidovudine levels	1.013 (0.015–3.4)			1.013 (0.015–3.4)	
Lamivudine Peritoneal to Plasma ratio	2.305 (0.625–4.181)			2.305 (0.625–4.181)	
Peritoneal equilibration test					
Lamivudine, n^a^	24	6	0.060^b^	30	
Plasma Lamivudine levels (2 h)	2.05 (0.589–5.14	0.024 (0.019–0.087)	0.055^c^	1.655 (0.022–3.8)	< 0.001
PD effluent Lamivudine levels (4 h)	0.803 (0.319–1.845	0.011 (0.003–0.019)	0.005^c^	0.446 (0.016–1.56)	
Lamivudine D/P ratio	0.640 (0.321–0.866)	0.476 (0.125–0.814)	0.478^c^	0.640 (0.299–0.844)	
Abacavir, n	20	6		26	
Plasma Abacavir levels (2 h)	4.13 (1.55–11.04)	7.62 (0.0039–27.60	0.952	4.13 (1.22–14.8)	0.424
PD effluents Abacavir levels (4 h)	4.61 (2.38–8.63)	2.31 (0.0032–10.20)	0.626	4.05 (2.23–9.08)	
Abacavir D/P ratio	1.264 (0.400–2.286)	0.612 (0.475–0.787)	0.279	0.689 (0.435–2.209)	
Efavirenz, n	19	5		24	
Plasma Efavirenz levels (2 h)	53 (30.9–77.5)	0.259 (0.046–83.9)	0.303	49.8 (29.95–80.7)	< 0.001
PD effluents Efavirenz levels (4 h)	2.37 (1.48–4.19)	0.016 (0.015–3.39)	0.188	2.185 (1.04–3.79)	
Efavirenz D/P ratio	0.050 (0.029–0.087)	0.061 (0.040–0.121)	0.456	0.050 (0.035–0.088)	

Abbreviations: D/P ratio, peritoneal dialysate to plasma ratio; HIV‐1 detectable, HI‐1 particles detected in PD effluents; PD, peritoneal dialysis.

^a^ Pearson's *χ* ^2^ test—Clinic and PET visit proportions of ARV drugs by HIV‐1 detectability in CAPD effluents.

^b^ Fisher's exact test—Clinic and PET visit ARV drug levels in HIV detectable (> 20 copies/mL) and undetectable (< 20 copies/mL) cohorts.

^c^ Two‐sample Wilcoxon rank‐sum (Mann‐Whitney) test—Clinic and PET visit ARV drug levels and D/P ratios in plasma and PD effluents by HIV‐1 detectability in CAPD effluents.

^d^ Wilcoxon signed rank test comparing drug levels in blood/plasma and PD effluents during clinic and PET visits.

**Figure 1:**
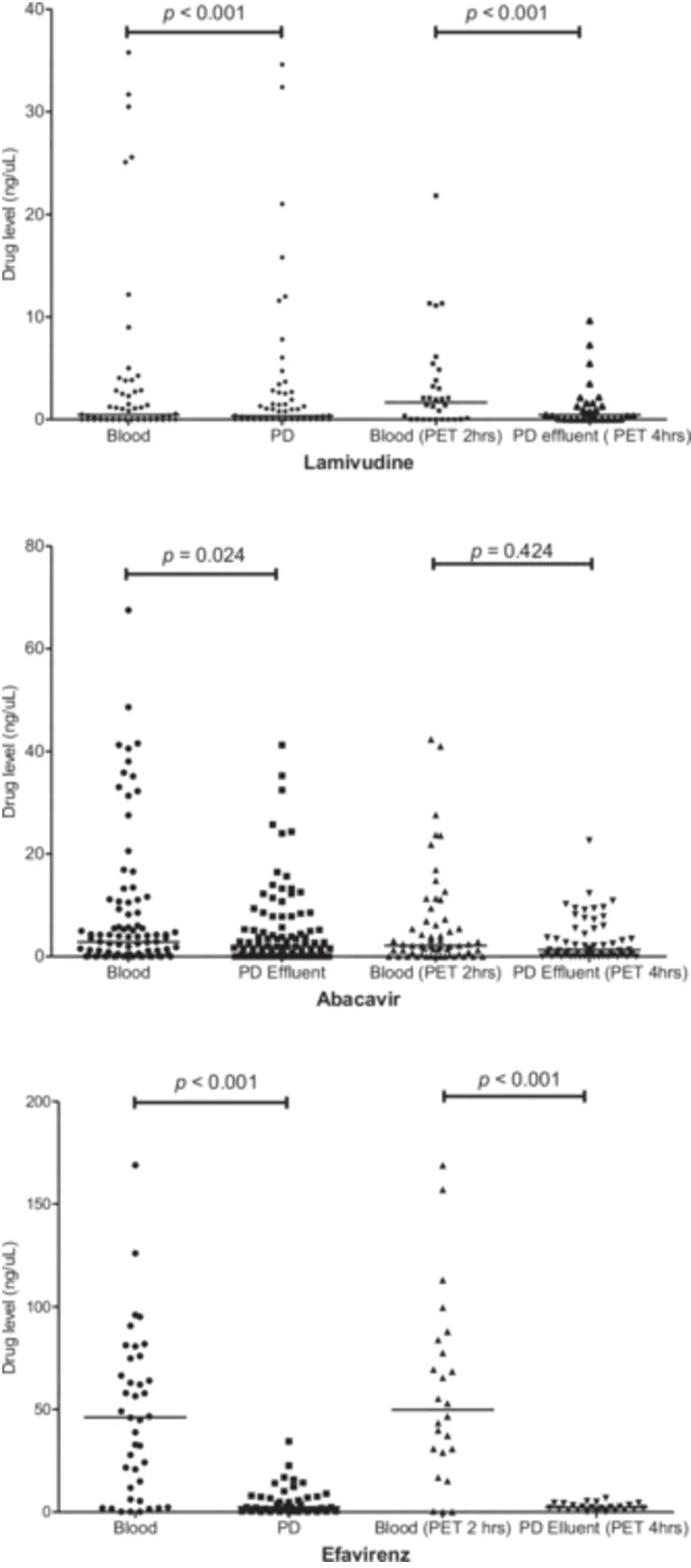
Scatter plot of antiretroviral drug levels in plasma and PD effluents during regular clinic visit and PET visit. Note: Wilcoxon signed rank test comparing drug levels in blood/plasma and PD effluents during clinic and PET visits.

The calculated peritoneal to plasma (D/P) ratios for drug concentrations measured during regular clinic visits for most of the ARV drugs were not significantly different among those with detectable and undetectable HIV‐1 in CAPD effluents, except for abacavir (1.717 vs. 0.644; *p* < 0.027) which had a significantly higher peritoneal to plasma ratio among those with detectable HIV‐1 in CAPD effluents, Table 2.

Figure 2 demonstrates the distribution of peritoneal‐to‐plasma (D/P) ratios for antiretroviral drugs measured during routine clinic visits and during the standardized 4‐h PET. Median D/P ratios for lamivudine (3TC), efavirenz (EFV), and abacavir (ABC) were generally comparable between clinic‐derived and PET‐derived measurements, with only lamivudine showing a statistically significant difference between the two approaches (*p* = 0.036). Furthermore, the IQR for abacavir (1.03 and 1.77, clinic and PET, respectively) and lopinavir (1.45) was very large.

**Figure 2:**
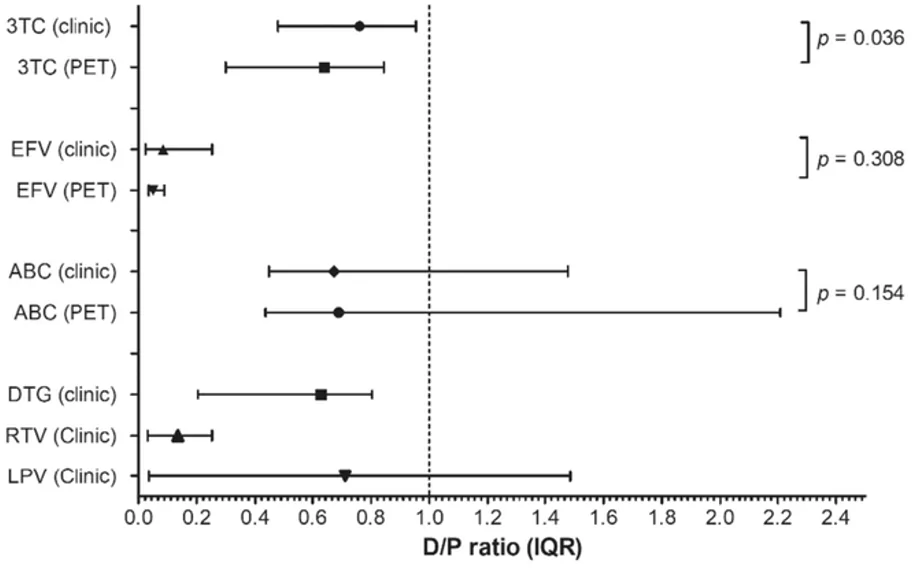
The interquartile range of antiretroviral drug levels versus D/P ratio measured during clinic and PET visits. Note: Wilcoxon signed rank test comparing interquartile ranges of drug levels during regular clinic and PET visits and their D/P ratios.

### 3.3 Spearman Rank Correlation Between HIV‐1 Shedding Into CAPD Effluents and ARV Drug Levels

On Spearman correlation analysis, blood levels of lamivudine (*p* = 0.003), efavirenz (*p* = 0.008), and abacavir (*p* = 0.035) were inversely correlated with HIV viral load in CAPD effluents during regular clinic visit, Table 3.

**Table 3:** Spearman's rank correlation between HIV‐1 detectable viral load in CAPD effluents and ARV drug levels.

Variables	Spearman's rank correlation coefficients (ρ)	*p*‐value
Lamivudine		
Clinic blood level	−0.380	0.003
Clinic PD level	−0.426	< 0.001
Peritoneal to Plasma ratio	−0.102	0.442
PET blood level	−0.358	0.052
PET PD 4 h level	−0.518	< 0.003
PET D/P ratio	−0.130	0.501
Efavirenz		
Clinic blood level	−0.405	0.008
Clinic PD level	−0.378	0.014
Peritoneal to Plasma ratio	−0.006	0.970
PET blood level	−0.223	0.295
PET PD 4 h level	−0.277	0.190
PET D/P ratio	0.181	0.397
Abacavir		
Clinic blood level	−0.290	0.035
Clinic PD level	−0.134	0.341
Peritoneal to Plasma ratio	0.330	0.019
PET blood level	−0.011	0.957
PET PD 4 h level	−0.077	0.707
PET D/P ratio	−0.186	0.374

Abbreviations: D/P ratio, dialysate to peritoneal ratio; PD, peritoneal dialysis; PET, peritoneal equilibrium/equilibration test.

## 4 Discussion

This prospective cross‐sectional study evaluated the ART drug penetration into the peritoneal compartment and its association with the detection of HIV‐1 in CAPD effluents. ART drug penetration was not found to be associated with the shedding of HIV‐1 into the peritoneal compartment. However, low plasma ART drugs concentrations were found among those with detectable HIV‐1 in CAPD effluents. Additionally, there was an inverse correlation between plasma drug concentration of lamivudine, efavirenz, and abacavir and the HIV‐1 viral load detectable in the CAPD effluents.

This study demonstrated a significant association of low plasma levels of lamivudine, and efavirenz with HIV‐1 shedding into CAPD effluents evaluated during regular clinic visits. This highlights a risk for the emergence of HIVDRMs, particularly non‐nucleotide reverse transcriptase inhibitors (NNRTIs) which can lead to ART failure [19]. In previous studies, suboptimal ART levels were reported as a consequence of poor adherence in persons failing ART [20, 21]. The group with HIV‐1 detectable on CAPD effluents had high plasma viral load and significantly low T‐cell CD4 and total white blood cell counts, implying poor HIV control despite being on ART for a long time. Our previous report in this population demonstrated a significant association of high HIV‐1 plasma levels with the presence of HIVDRMs which were likely contributed by poor ART adherence [16]. However, in this study, the maintenance of relatively acceptable urea and electrolytes levels indicates that there was no obvious non‐compliance with the CAPD procedure among those with detectable HIV‐1 in CAPD effluents compared to those who were undetectable.

The penetration of ARV drugs into the peritoneal compartment was approximated using drug concentration peritoneal‐to‐plasma ratios. The calculated ratios were above 60% for most ARV drugs tested, suggesting a relatively good peritoneal penetration, except for efavirenz and ritonavir, both having a median D/P ratio well below 10%. This is consistent with the literature on ritonavir and efavirenz, which are moderately bioavailable (~50% in plasma) following first‐pass hepatic metabolism, largely protein bound (~98%–99%), poorly distributed across the peritoneal membrane and largely eliminated by the liver into faeces. Moreover, ritonavir has a very negligible extraction ratio (1%) [6, 19, 20].

The D/P ratios for lamivudine was not found to be significantly different between participants with the detectable HIV‐1 in CAPD effluents compared to those with undetectable HIV‐1 RNA in the effluents; thus, suggesting a stable peritoneal compartment penetration among those with detectable and undetectable HIV‐1 in CAPD effluents. This correlates with our previous finding, which demonstrated comparable therapeutic exposure in “renally adjusted” lamivudine doses in blood and peritoneal compartments among participants with detectable and undetectable HIV‐1 RNA in CAPD effluents [18]. Furthermore, this likely indicates that sub‐therapeutic plasma levels and HIVDRMs are the predominant factors influencing the shedding of HIV‐1 in our population [19, 22]. The literature on lamivudine suggests high bioavailability (~86%) following oral admiration of a tablet and wide distribution throughout the body with a volume of distribution of 1.3 ± 0.4 L/kg, which is independent of dose and not correlated to BMI [9, 11, 21].

D/P ratios were highly variable, likely influenced by uncontrolled blood and CAPD effluent sampling relative to the dosing of the ARV drugs. However, differences in drug handling, which includes absorption, distribution, metabolism and elimination, pharmacogenomics, adherence consistency, and drug interactions with concomitant medications and food, are recognized contributors to inter‐and intra‐individual ARV drug variability [10]. Kidney failure and, dialysis‐related factors such as persistent inflammation, protein wasting syndrome and peritoneal membrane transport/characteristics, are other potential contributors to this variability [18, 22, 23]. Notably, our clinic is the only CAPD center for the Free State Province, with patients traveling vast distances of up to 400 km to reach this specialist center. This makes it difficult to control, monitor and time the dosing of the ARV drugs relative to the blood sampling in the clinic. Despite these challenges, plasma levels were still shown to be reliable predictors of breakthrough HIV‐1 particles contaminating the dialysis waste.

D/P ratios for abacavir and lopinavir/ritonavir suggests increased transport into or decreased efflux out of the peritoneal compartment among those with detectable HIV‐1 in effluents. Pharmacokinetic studies indicate that abacavir is 50% protein‐bound but has good penetration into compartments such as the cerebrospinal fluid (CSF) [5]. Abacavir concentration curves generated by Capparelli et al. [24, 25] shows a flatter CSF curve compared to plasma over time, reflecting slow influx and outflux in the CSF compartment. There is a dearth of abacavir pharmacokinetic studies in CAPD. However, it's likely that similar pharmacokinetic phenomena explain the observed D/P ratios in our study. For lopinavir/ritonavir, this phenomenon occurred in only one patient, which could be due to a unique, extensive metabolic profile that lead to high plasma drug levels and increased clearance of polar metabolites. Lymphatic drainage into peritoneum cavity may also be a transporter of non‐polar metabolites/pro‐drug [20].

Median D/P ratios for efavirenz and abacavir were comparable between clinic‐ and PET‐derived measurements. While lamivudine showed mild discordance, its estimates remained reasonably aligned. This overall concordance suggests that clinic‐derived measurements provide a practical, robust surrogate for assessing peritoneal drug penetration, bypassing the complexity of PET procedures. Unless PET‐derived estimates can be shown to improve prediction of tissue drug exposure, viral suppression or other clinical outcomes, the added complexity and collection challenges associated with PET sampling may not be justified for routine compartment penetration assessments. Although PET‐based D/P ratios offer a theoretically more direct assessment of drug distribution within the target compartment, their utility in this dataset may have been limited by the lack of control over the timing of drug administration relative to sampling. Nevertheless, despite this limitation in both sampling techniques, the clinic‐derived estimates of serum drug levels and D/P ratios seem to offer relatively good correlation to meaningful outcomes of PD effluent HIV viral particle contamination and peritoneal compartment drug exposure.

It is noteworthy that only one patient on dolutegravir had detectable HIV‐1 in effluents out of 11 patients. This may be a poor drug‐adherence issue. Adherence to medications is a known problem in ESKF patients, not only to ART but also to the CAPD procedure, which is more cumbersome [26]. Therapeutic dose monitoring (TDM) was not specifically evaluated in this study. However, inadequate exposure to antiretroviral drugs is suggested to contribute to the shedding of HIV particles into CAPD effluents, which suggests a role for TDM in CAPD patients with unsuppressed viral load. Furthermore, TDM has been suggested to be an objective method to monitor adherence [21, 27], suggesting a role for TDM in regular surveillance for this outcome.

This study has several limitations. Firstly, it is a small single‐center study, which inherently limits the generalizability of the findings. However, in this regard, it is strengthened to some extent by its prospective design. Secondly, ART adherence was not specifically monitored. Adherence has several barriers and may be a contributor to variable drug levels in our study population. Further, drug testing during regular clinic visits was not timed in relation to taking the ARV drug. However, as discussed, some of the derived results seem to have been robust despite this handicap.

## 5 Conclusions

This study demonstrated an association of low plasma lamivudine, efavirenz, and abacavir levels with HIV‐1 shedding into CAPD effluents, with these drugs' plasma concentrations inversely correlated with the detectable HIV viral load in CAPD effluents. The results of the study highlight the possible value of TDM during regular clinic visits to identify patients at risk of therapeutic failure and contaminating CAPD effluents with HIV‐1 particles. However, this merits further research to understand the influence of CAPD on ART pharmacokinetics in ESKF patients shedding HIV‐1 particles in CAPD effluents.

## Author Contributions

Teboho Mooko collected and analyzed the data, and contributed majorly to manuscript writing. Kwazi Ndlovu and all other co‐authors supervised Teboho Mooko and supported the project. Conceptualization: Teboho Mooko, Feziwe Bisiwe, Martin Nyaga, Molefi Morobadi, Perpetual Chikobvu, Thabiso Mofokeng, Gabre Kemp, Dominique Goedhals, and Kwazi Ndlovu. Data curation: Teboho Mooko and Kwazi Ndlovu. Formal analysis: Teboho Mooko, Perpetual Chikobvu, Gabre Kemp, and Kwazi Ndlovu. Funding acquisition: Teboho Mooko, Thabiso Mofokeng, Martin Nyaga, Molefi Morobadi, Gabre Kemp, Dominique Goedhals, and Kwazi Ndlovu. Investigation: Teboho Mooko, Gabre Kemp, and Kwazi Ndlovu. Methodology: Teboho Mooko, Morobadi, Feziwe Bisiwe, Dominique Goedhals, Gabre Kemp, and Kwazi Ndlovu. Project administration: Teboho Mooko and Kwazi Ndlovu. Resources: Feziwe Bisiwe, Morobadi, Martin Nyaga, Molefi Morobadi, Thabiso Mofokeng, Dominique Goedhals, and Gabre Kemp. Supervision: Feziwe Bisiwe, Morobadi, Martin Nyaga, Molefi Morobadi, Perpetual Chikobvu, Thabiso Mofokeng, Gabre Kemp, Dominique Goedhals, and Kwazi Ndlovu.

## Ethics Statement

The SHIPPeD study protocol was approved by the University of the Free State‐Health Sciences Research Ethics Committee (UFS‐HSD2020/0318/2710 & UFS‐HSD2021/0267/2505) and permission for the study was granted by the Free State Department of Health.

## Consent

All participants provided written consent before enrollment.

## Conflicts of Interest

The authors declare no conflicts of interest.

## Supporting information


Supporting File

## Data Availability

The data that support the findings of this study are available on request from the corresponding author. The data are not publicly available due to privacy or ethical restrictions. The data underlying this article are available in the article and in its online supporting material.
