# Supplementary material for: The Influence of Plasma and Peritoneal Antiretroviral Drugs Levels on the Shedding of HIV‐1 Into Peritoneal Dialysis Effluent
Source: J Med Virol. 2026 Oct 24;98(10):e71192. doi: 10.1002/jmv.71192 (PMC13695467; doi:10.1002/jmv.71192)
Supplement: Supplementary file 1 [file JMV-98-e71192-s001.docx]

**Supplementary Information on peritoneal equilibrium test procedure and sample collection (S1).**

***PET***

***Peritoneal Equilibrium Test***

- *The PET determines the membrane transport type of the patient (based on D/P* ***creatinine****) and ultrafiltration capacity.*

*The ultrafiltration capacity helps to define ultrafiltration failure and at 4 hours should be: -*

*2L 2.5% > 200 mL*

*2L 4.25% > 400 mL*

- PET is done as a 4-hour test
- Need 2,5% or 4.25% Dianeal bag dwelling for 4 hours

TEST Procedure:

Infuse Dianeal 2.5% or 4.25% (Irrelevant of what current Prescription is) keep bag connected.

Note down the time: 0 Hour

- **Dialysate Sample 1** (0Hour): Drain 200ml in bag with port. Take 10 ml sample infuse back in patient

 Creatinine and Glucose (optional)

- **Dialysate Sample 2** (2h-120min): Drain 200ml in fill bag with medicine port draw 10ml sample. Give fluid back to patient, keep bag connected still.

 Draw serum for Creatinine and Glucose

- **Blood/Serum Sample** (120min)

 Creatinine and Glucose

- **Dialysate Sample 3 (**4h-240min Drain bag in fill bag with medicine port draw 10ml sample. Give fluid back to patient. Connect new bag and fill.

 Creatinine and Glucose

- Record volume drained after 4 Hours

**FORMULA:**

**Dialisate Creatinine / Serum Creatinine] =>TOTAL X 100%**

**High/Fast Transporter	0.82 – 1.03**

**High average Transporter	0.65 – 0.81**

**Low average Transporter	0.50 – 0.64**

**Low Transporter	0.34 – 0.49**

**Supplementary Information on liquid chromatography conditions:**

**Supplementary Figure 1 (S2):** Liquid Chromatography separation of seven renal friendly ART in plasma and PD effluents.

**Supplementary Figure 1 (S1):** Retention time (minutes) for each analyte separated on a C18 (50mm x 2.0mm, Aqua, Phenomenex) column at a flow rate of 300 uL/minute using a 4 minutes gradient from 45% solvent A (H2O/0.1% formic acid) to 95% solvent B (MeOH/0.1% formic acid) with a total run time of 15 minutes to allow for column re-equilibration.

**Mass Spectrometry analysis and conditions**

The eluted analytes were ionised by electrospray in the TurboV ion source with 500 ^o^C heater temperature to evaporate excess solvent, 40 psi nebuliser gas, 30 psi heater gas and 15 psi curtain gas. The ion spray voltage was set at 5500 V. Samples were analysed in positive ionization mode. The targeted analyses were performed using 2 MRM (multiple reaction monitoring) transitions per analyte. The peak area on the chromatogram generated from the first and most sensitive transition was used as the quantifier while the second transition is used as a qualifier. The qualifier serves as an additional level of confirmation for the presence of the analyte, the retention time for these two transitions needs to be the same. The peak areas of the unknown samples were related back to a quantified value using the calibration curve for each analyte. All data acquisition and processing were performed using Analyst 1.5 software (AB Sciex, Framingham, MA, USA). The mass-to-charge (m/z) ratio of each ARV standard drug/fragment is summarised in S2-Table 1:

**Supplementary Table 1 (S2):** The mass-to-charge (M/Z) ratio of seven renal friendly ARV drugs in serum and PD effluent matrix.

| **Positive ionization mode** | | **Analyte** |
| --- | --- | --- |
| **Q1 (m/z)** | **Q3 (m/z)** |  |
| 268.0 | 127.1 | Zidovudine (ZID1) |
| 268.0 | 110.1 | Zidovudine (ZID2) |
| 230.0 | 112.1 | Lamivudine (LAM1) |
| 230.0 | 94.6 | Lamivudine (LAM2) |
| 629.2 | 155.1 | Lopinavir (LOP1) |
| 629.2 | 447.3 | Lopinavir (LOP2) |
| 287.1 | 191.1 | Abacavir (ABA1) |
| 287.1 | 79.1 | Abacavir (ABA2) |
| 316.0 | 244.1 | Efavirenz (EFA1) |
| 316.0 | 272.1 | Efavirenz (EFA2) |
| 419.9 | 277.1 | Dolutegravir (DOL1) |
| 419.9 | 277.1 | Dolutegravir (DOL2) |
| 721.1 | 140.0 | Ritonavir (RIT1) |
| 721.1 | 296.1 | Ritonavir (RIT2) |

M/Z ratio, Mass-to-Charge ratio.
